# Assessing the usability of aromatic mouthwashes in alleviating physiological stress responses

**DOI:** 10.3389/froh.2024.1343937

**Published:** 2024-04-04

**Authors:** E. A. Chayani Dilrukshi, Tatsuki Ogino, Mami Ishikawa, Hiroki Kuroda, Shusaku Nomura

**Affiliations:** ^1^ Graduate School of Engineering, Nagaoka University of Technology, Nagaoka, Niigata, Japan; ^2^ Department of Industrial Management, Faculty of Applied Sciences, Wayamba University of Sri Lanka, Kuliyapitiya, Sri Lanka; ^3^ R&D, Sunstar Inc., Takatsuki, Osaka, Japan

**Keywords:** aroma, mouthwash, bio-signals, heart rate, subjective impressions, physiological stress response, oral care

## Abstract

**Background:**

Mouthwashes play a pivotal role in oral care, and their efficacy has been explored extensively across various dimensions. As a contribution to the development of novel oral care products, this study aims to investigate the psychophysiological effects of aromatic mouthwashes during the resilience period from a short-term cognitive stressor utilizing biological signals and subjective evaluations.

**Methods:**

A within-participant experimental design with 22 healthy females was conducted with four mouthwashes; peppermint (Mint), peppermint + bergamot (MB), peppermint + sweet orange (MO), and peppermint + lavender (ML), and water as the control (Ctl), after a 20-min calculation task. Subjective evaluations and physiological responses including skin conductance level and electrocardiogram were recorded throughout the experiment.

**Results:**

Citrus mouthwashes (MO and MB) showed a greater decrease in heart rate and a significant increase in the high-frequency component of heart rate variability. The participants indicated a significant effect in terms of “flavor preference” and “refreshing sensation” for mouthwash use compared to the Ctl.

**Conclusion:**

The results suggest that rinsing with citrus-flavored mouthwashes has a positive impact in alleviating the physiological stress response (in terms of cardiac activity). These findings may have implications for the development of innovative, novel oral care products that promote stress reduction and improve oral health.

## 1 Introduction

Bio-signals, measured via human-machine interaction are indispensable for detecting acute stress and are broadly categorized into physical and physiological signals (1). Physical bio-signals encompass measurements of bodily deformations arising from muscle activities, including pupil size, eye movements, blinks, and various postures and movements. Physiological bio-signals are more directly connected to essential bodily functions, involving cardiac activities [electrocardiogram (ECG), blood volume pulse], brain operations (electroencephalogram), exocrine activities [evaluated through electrodermal activity (EDA)], and muscle excitability gauged via electromyography (2). EDA also referred to as galvanic skin response (GSR) or skin conductance (SC), manifests because of constant changes in the skin's electrical property fluctuations caused by sweating. This dynamic electrical change, measurable through placing electrodes on the skin, offers insights into emotional, psychological, or physiological arousal (3). Heart rate (HR) stands as the extensively utilized and direct metric for assessing acute stress, and heart rate variability (HRV), indicating fluctuations in time intervals between successive heartbeats (4–6), serves as a non-invasive tool for assessing psychological states like fatigue, stress, and anxiety (7). Numerous studies across psychology and neuroscience have affirmed that HRV measurements reflect the continuous interaction between the sympathetic and parasympathetic branches of the autonomic nervous system (ANS) (8, 9).

Research has extensively employed various bio-signals to evaluate acute stress response. Notably, studies utilizing ECG and SC have shown promising results (10–12). For instance, a study found that listening to music before a standardized stressor affected the ANS (in terms of a faster recovery), with an increase in HR during the psychosocial stress test followed by a decrease in HR in the resilience period (13). Another study indicated that olfactory stimulation by Hinoki cypress leaf oil significantly increased the high frequency (HF) component of HRV, inducing physiological relaxation (14). Additionally, a study on citrus mint-flavored mouthwash showed a significant decrease in skin conductance level (SCL) during the resilience period, after a calculation task (15). These findings underscore the potential of ECG and SCL in assessing acute stress responses.

Mouthwashes, integral to oral care, are liquid products designed to enhance oral hygiene. These solutions are used by individuals to supplement their regular dental care routines, targeting various oral concerns such as bad breath, bacteria control, and gum health (16, 17). They are formulated with active ingredients, including antimicrobial agents and fluoride, alongside flavoring agents like menthol, mint, citrus, or herbal extracts, providing a refreshing sensation (18). In this context, studies evaluated the efficacy of various mouthwash formulations, aiming to establish their effects on oral health and well-being. Research demonstrated that mouthwash rinsing holds significant potential in alleviating xerostomia, ameliorating dry mouth symptoms, and enhancing salivary production (19–21), preventing dental caries (22, 23), addressing dentinal sensitivity (24), and inhibiting the buildup of plaque and the development of gingivitis (25–27).

Conversely, concerning aromatherapy, many studies have investigated the psychophysiological effects of different aromas. Lavender alleviated job-related stress in nurses (28), bergamot essential oil exerted psychological and physiological effects in a relatively short time (29), and peppermint oil was found to be beneficial in relieving stress (30) and enhancing sleep quality (31). Our previous study suggested that inhalation of orange essential oil inhibited the physiological stress response (32). Given that these aroma studies have demonstrated diverse psychophysiological efficacies of aromas on ANS, it is reasonable to anticipate a certain level of psychophysiological impact from the utilization of aromatic mouthwashes.

Aromatic mouthwashes, a subset integrating sensory experiences with oral hygiene, not only offer functional benefits but also sensory pleasures. However, research about the effects of aromatic mouthwashes remains relatively limited. One study examined an aromatic oral care solution incorporating peppermint, tea tree, and lemon essential oils, and found its potential to enhance the oral health of elderly dementia patients within long-term care settings (33). Another study reported that an aroma gargle comprising peppermint, lemon, tea tree, and ylang-ylang was effective in relieving stress, decreasing xerostomia, and reducing the objective halitosis of the nurses (34).

Despite extensive research on mouthwash efficacy, few studies have evaluated their impact through bio-signals. One study suggested that mouthwashes, carbohydrates, or a placebo, improve running endurance compared to the control condition in a heat-stress environment, with no significant difference in HR, rectal temperature, skin temperature, or skin blood flow between the three trials (35). Jeffries et al. (36) showed that the L-menthol mouth rinse improved the performance of athletes during exercise compared to the placebo, whilst there were no significant differences found in bio-signals such as HR and skin temperature (36). Another study that investigated the physiological effects of a mint-flavored mouthwash at bedtime to enhance subsequent sleep quality using a wristwatch-type HR monitoring device found that the use of mouthwashes before sleep promotes a positive mood and potentially improves physiological sleep (37). Moreover, studies that focused on the physiological effects of citrus mint flavored mouthwash (15), and mouthwashes with varying alcohol concentrations (38), revealed a significant decrease in SCL during the resilience phase after a cognitive stressor. Another study utilizing mouthwashes with different types of binders (thickening agents) suggested that with higher thickness levels, an increased HF component of HRV can be exhibited with a potential recovery from the physiological stress response (39, 40).

So far, previous studies have evaluated the efficacy of different mouthwash formulations for various purposes, and some studies have explored the usage of mouthwashes utilizing bio-signals. However, the exploration of aroma-infused mouthwashes in the resilience period after a short-term stressor using biological signals has not been extensively pursued. Addressing this gap, the current study strives to contribute to the development of innovative oral care products by examining the psychophysiological effects of aromatic mouthwashes during the resilience period following a short-term cognitive stressor. The research hypothesis posits that the administration of aromatic mouthwashes will alleviate physiological stress responses within the ANS. Specifically, during the resilience phase, it is anticipated to observe a greater decrease in HR and SCL compared to the mouthwashes without aroma, indicative of a sympathetic nervous system (SNS) response. Simultaneously, it is hypothesized that there will be a greater increase in the HF component of HRV, reflecting a parasympathetic nervous system (PSNS) response.

Following the working hypothesis, as a part of new oral care product development, this research aims to investigate the psychophysiological potential of aromatic mouthwashes incorporating peppermint, lavender, bergamot, and orange aromas in alleviating acute stress response, utilizing an array of biological signals including HR, HRV, and SCL. The research methodology of the study employed a within-participant experimental design involving 22 females in a laboratory setting. The experimental conditions included four different types of mouthwash, and water served as the control (Ctl) condition. Biological signals, including HR, SCL, and HRV, were assessed to evaluate the impact of aromatic mouthwashes on the ANS during the resilience period.

## 2 Materials and methods

### 2.1 Participants

Twenty-two healthy female university students with a mean [±standard deviation (SD)] age of 22.32 (±0.92) years participated in the study. All participants had normal gustation, and based on their self-reports, none of them had a history of gustatory dysfunction. The study excluded the participants who were habitual smokers. The participants were provided with compensation in the form of monetary incentives for their involvement in the study, aiming to acknowledge and reciprocate their contribution to the research endeavor. They were instructed to abstain from consuming food, beverages (except water), vigorous physical activities, or oral care routines for 1 h before each experimental trial. Detailed prior information regarding the exact composition of the mouthwashes was not provided to the participants.

The research adhered to the ethical principles outlined in the Helsinki Declaration. Informed consent was obtained from all participants before their involvement. The experimental design was approved by the ethics review committee of the Nagaoka University of Technology.

### 2.2 Experimental protocol

**Figure 1:**
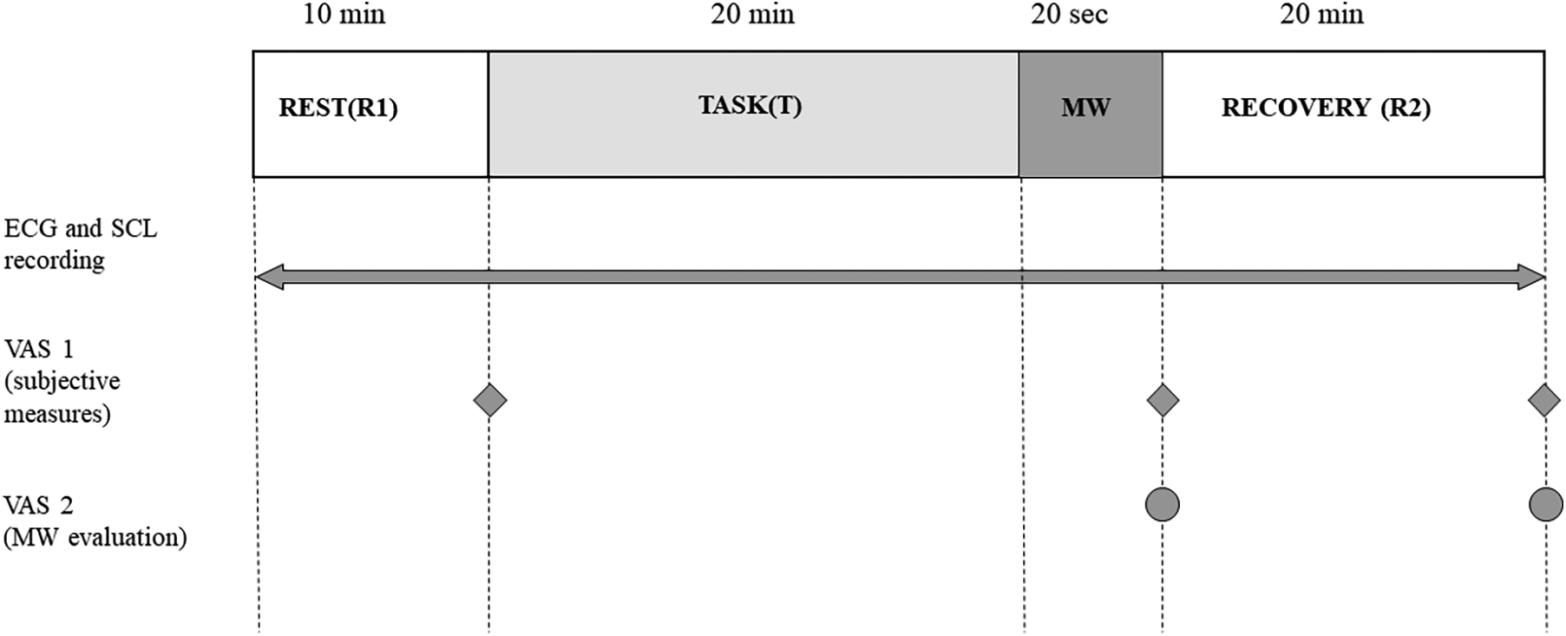
Schematic diagram of the experimental design. MW, rinsing with the mouthwash; ECG, electrocardiogram; SCL, skin conductance level; VAS, visual analog scale.

The study employed a within-participant experimental design, including an initial 10-min period of rest (referred to as R1), followed by a 20-min calculation task (T), a 20-s mouthwash rinsing period (MW), and a subsequent resilience period for 20 min (R2) as shown in Figure 1.

The experiment incorporated four distinct aromatic mouthwashes: peppermint (Mint), peppermint + bergamot (MB), peppermint + sweet orange (MO), and peppermint + lavender (ML), and mineral water with pH value 7.6 was used as the Ctl condition. Each participant was instructed to rinse their mouth for 20 s using 10 ml of the designated mouthwash or water. After rinsing, participants were directed to spit out the mouthwash into a paper cup. The participants received the mouthwashes in a counterbalanced sequence over five separate days, with all sessions conducted at approximately the same time of day.

All experimental procedures were conducted within an air-controlled laboratory environment, maintaining an average temperature of 23.68°C (±2.12°C) and a humidity level of 51.30% (±6.13%).

#### 2.2.1 Cognitive stressor

The calculation task, referred to as the Kraepelin test was used as the cognitive stressor in the study. It involved performing continuous mental calculations that consisted of adding single-digit numbers displayed on a computer screen. Participants were instructed to complete this task with both speed and accuracy. The Kraepelin test is a standardized cognitive stressor commonly utilized to evaluate participants' concentration and mental workload interacting with the computer (15, 32, 39, 40).

### 2.3 Mouthwashes formula

The experiment utilized four types of non-alcoholic mouthwashes with a similar general composition. These mouthwashes were formulated with components such as a surfactant (to enhance dispersion within the oral cavity), glycerine, and other typical ingredients commonly found in mouthwash products.

Each of these four mouthwash variants was prepared by infusing an aroma (Mint, MB, MO, and ML) into the basal mouthwash at a concentration of 0.1%. The specific composition of the aroma blend is detailed in Table 1. Peppermint essential oil base was utilized in all mouthwash formulations as it is an essential prerequisite in oral care product development attributing to its beneficial effects on oral health and overall sensory experience. The aromatic compositions, including specific aromas, their corresponding concentrations, and textures, were developed by expert aroma practitioners exclusively for research purposes.

**Table 1:** The percentage of blended essential oils used in the study.

	Mint	MB	MO	ML
Peppermint (*Mentha arvensis*)	40	40	40	40
l-menthol	30	30	30	30
Essential oil	0	10	15	5
Propylene glycol	30	20	15	25

All the values given in the table are denoted in percentages (%). Mint, peppermint; MB, peppermint + bergamot; MO, peppermint + sweet orange; ML, peppermint + lavender.

### 2.4 Measurements

#### 2.4.1 Physiological measurements

Throughout the experiment (R1-T-MW-R2; as illustrated in Figure 1), ECG and SCL measurements were recorded using a bio-amplifier (MP 150, BIOPAC Systems Inc., USA) operating at a sampling rate of 500 Hz and a resolution of 16 bits. For ECG measurements, electrodes were placed beneath the right clavicle and on the lower left abdomen, following the “Lead II” configuration (ECG sensor: TSD155C, BIOPAC Systems Inc., USA). For SCL measurements, electrodes were attached to the palmar side of the middle phalanges of the second and fourth fingers of the participant's non-dominant hand (sensor: TSD203, BIOPAC Systems Inc., USA). The acquired signals underwent filtration processes, including a 35 Hz low-pass pulse, a 50 Hz notch filter, and a 0.05 Hz low-cut filter for ECG. In the case of SCL, a low-pass filter set at 10 Hz was applied.

ECG data were collected for the analysis of both HR and HRV. HRV specifically focused on the high-frequency (HF) component within the frequency range of 0.15–0.40 Hz. This HF component serves as an indicator of activity within the cardiac PSNS (4).

Conversely, SCL measurements were employed as an indicator of SNS activity at peripheral sites and can reflect changes in physiological arousal or responses to stress (41).

#### 2.4.2 Psychological measurements

The participants' subjective evaluations were assessed using a visual analog scale (VAS 1) comprising five attributes: “fatigue”, “nervousness”, “refreshing feeling”, “drowsiness”, and “relaxation”. This assessment was conducted three times: after R1, following the MW, and after R2 as depicted in Figure 1. Additionally, a VAS 2 questionnaire was administered to determine preference among the mouthwashes after MW and at the end of R2. This questionnaire consisted of four items: “flavor preference”, “stimulative sensation (tingling sensation)”, “cooling sensation”, and “refreshing sensation”. In the VAS, a calibrated line was presented with endpoints at 0% and 100%, instructing participants to mark their perception on the line corresponding to each attribute based on their experience (39, 40, 42).

### 2.5 Data analysis

The raw data for HR, HF, and SCL were standardized (converted to *z*-score) to account for individual variations across participants and conditions. Based on the study hypothesis, the mean difference during R2 was calculated for each physiological parameter as the variable for analysis. The Shapiro-Wilk test was conducted to assess the normality of the data distribution. Given that the datasets followed a normal distribution, a repeated measure one-way analysis of variance (ANOVA) followed by Dunnett's *post-hoc* tests were performed to compare the effects of five conditions on the physiological parameters. Additionally, a two-way ANOVA was employed to assess the effect of both time and conditions on the psychological parameters. The statistical significance level for *p* was set at 0.05. The statistical analysis was carried out using R software (Version 4.2.2, R Foundation for Statistical Computing, Vienna, Austria).

## 3 Results

### 3.1 Physiological assessment

#### 3.1.1 Heart rate and heart rate variability

**Figure 2:**
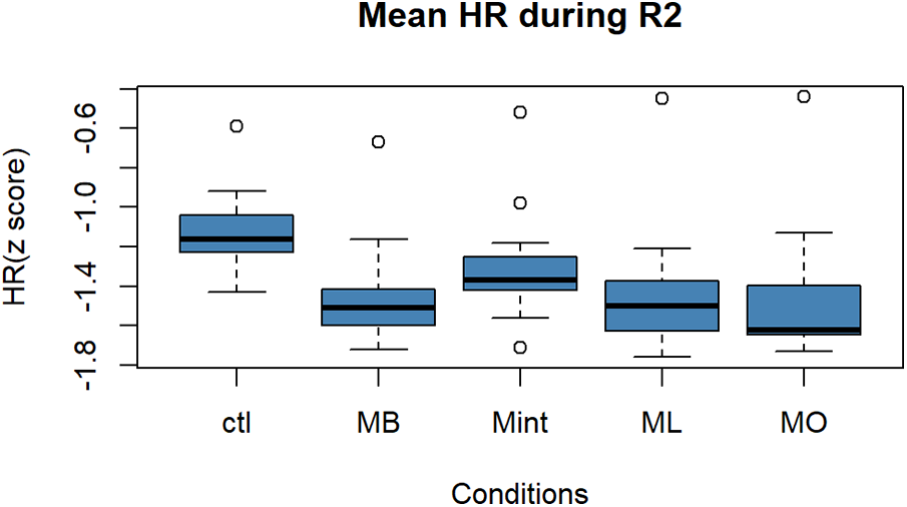
Box plot for the changes in the mean value of HR (*z* score) during R2 for all five conditions (*n* = 22). Within each box, horizontal black lines denote median values; boxes extend from the first and third quartile; vertical extending lines denote minimum and maximum values without outliers; dots denote the outliers. HR, heart rate; R2, recovery period; Ctl, control condition; MB, peppermint + bergamot; Mint, peppermint; ML, peppermint + lavender; MO, peppermint + sweet orange.

**Figure 3:**
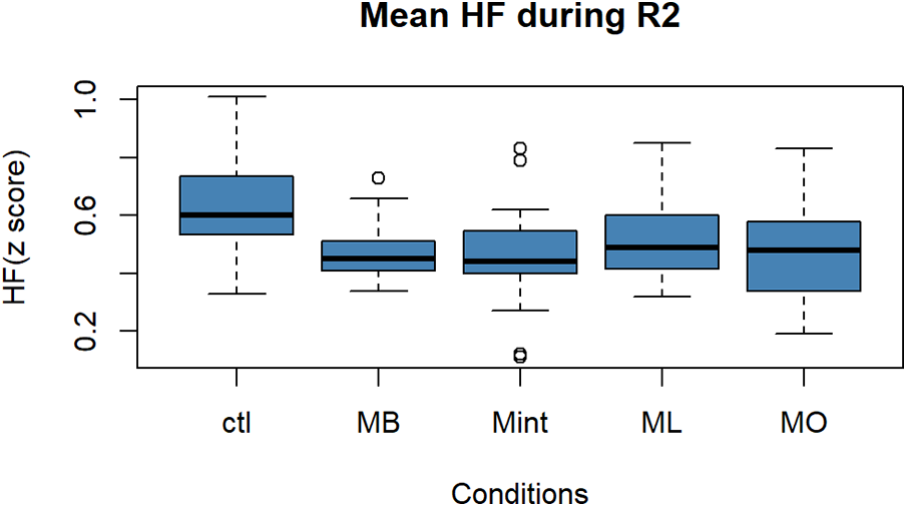
Box plot for the changes in the mean value of the HF component of HRV (*z* score) during R2 for all five conditions (*n* = 22). HF, high-frequency component of heart rate variability; R2, recovery period; Ctl, control condition; MB, peppermint + bergamot; Mint, peppermint; ML, peppermint + lavender; MO, peppermint + sweet orange.

Figures 2, 3 show the box plots for the mean value of the HR and HF components of HRV during the resilience period, R2, respectively. In Figure 2, a one-way ANOVA showed that there was a statistically significant difference in mean HR value between the conditions [*F* (4, 90) = 5.923, *p* < 0.001]. Performing *post hoc* analysis, MB, ML, and MO mouthwashes exhibited a significantly greater decrease in HR compared to Ctl (vs. MB: *p* < 0.001, 95% CI: [−0.53, −0.12], vs. ML: *p* = 0.0008, 95% CI: [−0.53, −0.11], vs. MO: *p* < 0.001, 95% CI: [−0.54, −0.12]).

As for HF in Figure 3, the ANOVA revealed that there was a significant difference among the five conditions in the HF component of HRV [*F* (4, 90) = 3.246, *p* = 0.015]. Performing *post hoc* analysis, Mint, MB, and MO mouthwashes showed a statistically significant increase in HF compared to Ctl (vs. Mint: *p* = 0.014, 95% CI: [−0.29, −0.25], vs. MB: *p* = 0.012, 95% CI: [−0.29, −0.28], vs. MO: *p* = 0.02, 95% CI: [−0.28, −0.18]).

#### 3.1.2 Skin conductance level

**Figure 4:**
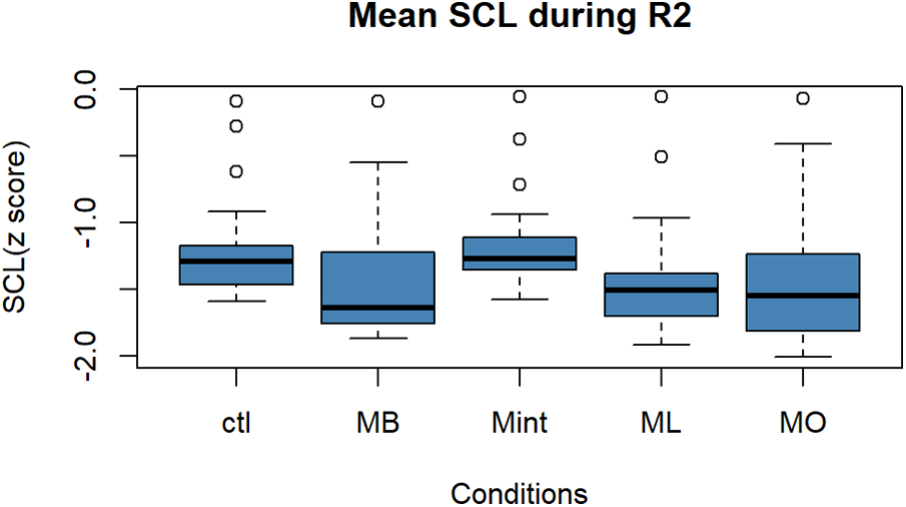
Box plot for the changes in the mean value of SCL (*z* score) during R2 for all five conditions (*n* = 22). SCL, skin conductance level; R2, recovery period; Ctl, control condition; MB, peppermint + bergamot; Mint, peppermint; ML, peppermint + lavender; MO, peppermint + sweet orange.

Figure 4 depicts the box plot diagram for the changes in the average SCL during the R2. There was no significant difference among the five conditions in terms of SCL during R2 [*F* (4, 90) = 1.6, *p* = 0.18].

### 3.2 Psychological assessment

As per the results of VAS 1, a two-way ANOVA revealed that there was no statistically significant difference between the conditions in any VAS score. Regardless of the experimental conditions, there was a statistically significant increase in VAS scores from after R1 to after MW (*p* < 0.01—*p* < 0.001; since the statistical results are extensive, the details will be omitted). This indicates that the computational task introduced in this study functioned as a short-term stressor (32, 37).

**Figure 5:**
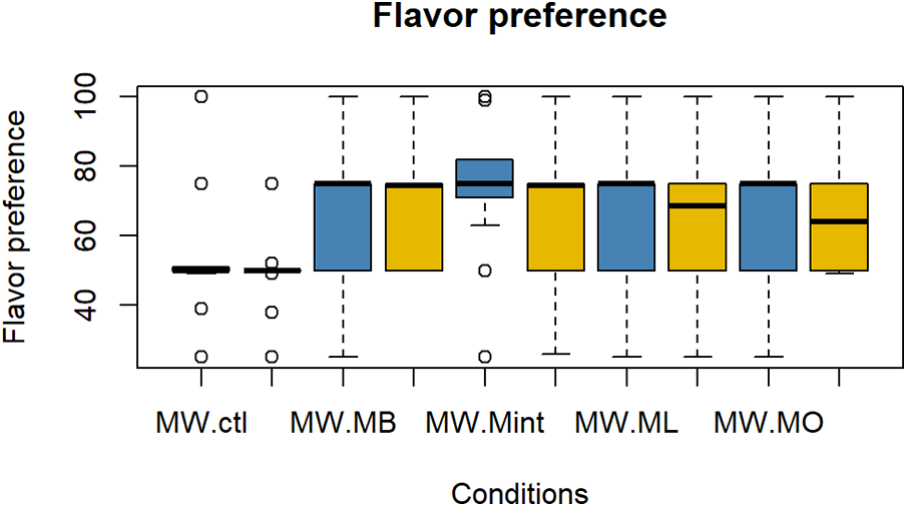
Box plot for the VAS scores of “flavor preference” for all five conditions (*n* = 22). MW, mouthwash administration; R2, recovery period; blue color: after MW, Yellow color: after R2.

**Figure 6:**
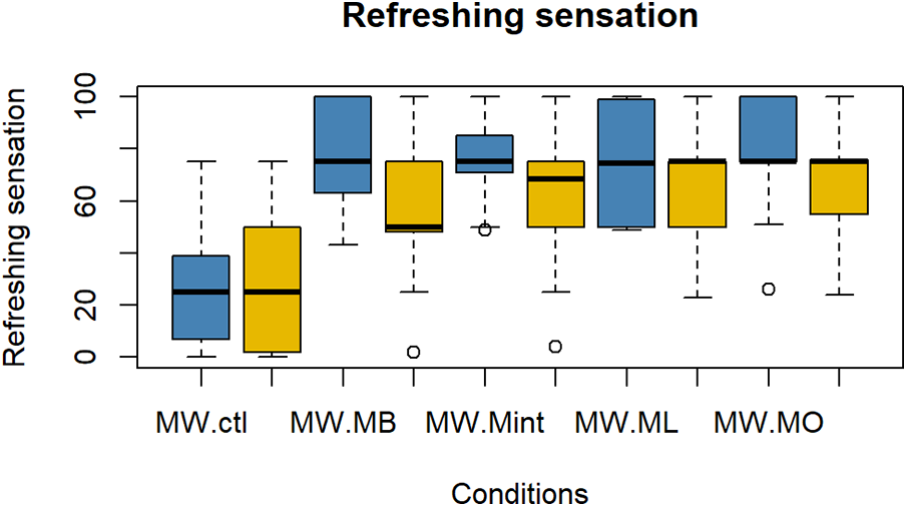
Box plot for the VAS scores of “refreshing sensations” for all five conditions (*n* = 22). MW, mouthwash administration; blue color: after MW, Yellow color: after R2.

**Figure 7:**
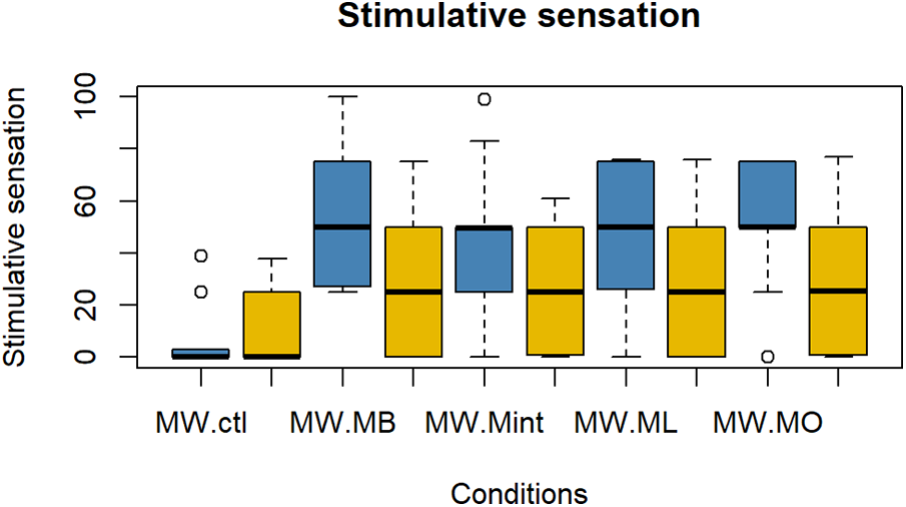
Box plot for the VAS scores of “refreshing sensations” for all five conditions (*n* = 22). MW, mouthwash administration; blue color: after MW, Yellow color: after R2.

**Figure 8:**
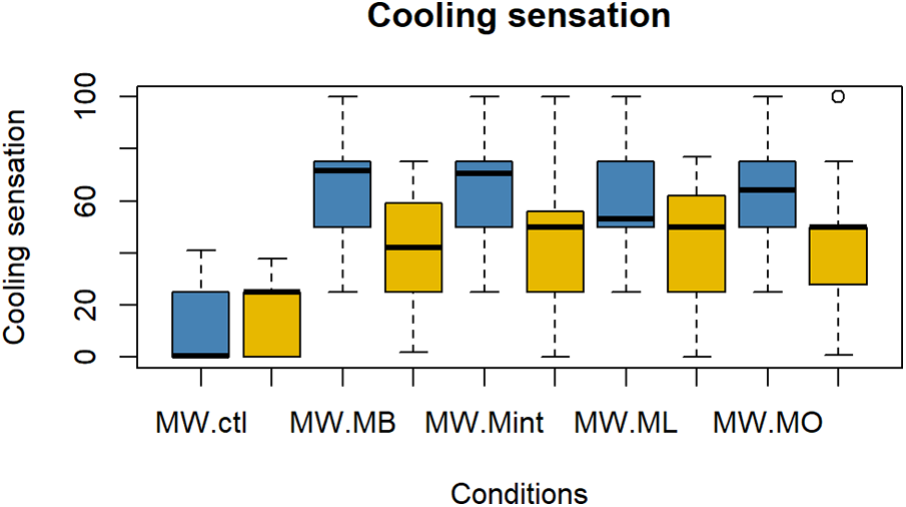
Box plot for the VAS scores of “cooling sensation” for all five conditions (*n* = 22). MW, mouthwash administration; blue color: after MW, Yellow color: after R2.

The evaluation results of subjective impressions for each condition (VAS 2) are given in Figures 5–8. Table 2 summarizes the statistical results of VAS 2. According to the results, there was no significant interaction between time × condition [*F* (4, 210) = 0.42, *p* = 0.79], and a significant effect between the conditions [*F* (4, 210) = 9.102, *p* < 0.001] was found for “flavor preference”. The *post hoc* test revealed significant differences among all the mouthwashes compared to the Ctl as given in Table 2. For “refreshing sensation”, there was no significant interaction between time × condition [*F* (4, 210) = 1.32, *p* = 0.26], and a significant effect between conditions [*F* (4, 210) = 37.69, *p* < 0.001], and the time [*F* (1, 210) = 13.51, *p* < 0.001] was found. The *post hoc* test revealed significant differences among all the mouthwashes compared to the Ctl, and a significant decrease in the “refreshing sensation” score in the period of R2. There was a significant interaction between time × condition [*F* (4, 210) = 2.54, *p* = 0.04], and a significant effect of condition [*F* (4, 210) = 18.55, *p* < 0.001], and the time [*F* (1, 210) = 29.07, *p* < 0.001] for “stimulative sensation”. The *post hoc* test revealed significant differences among all the mouthwashes compared to the Ctl, and a significant decrease in “stimulative sensation” in the period of R2. Furthermore, there was a significant interaction between time × condition [*F* (4, 210) = 2.57, *p* = 0.03], a significant effect of condition [*F* (4, 210) = 32.62, *p* < 0.001], and the time [*F* (1, 210) = 28.36, *p* < 0.001] for “cooling sensation”. The *post hoc* test revealed significant differences among all the mouthwashes compared to the Ctl, and a significant decrease in the “cooling sensation” score in the period of R2.

**Table 2:** The summary of two-way ANOVA results of VAS 2.

VAS item		After MW		After rest 2		Tests of between time periods
		Mean	SD	Mean	SD	
Flavor preference	Ctl	52.90	18.01	49.50	8.14	*F* (4, 210) = 2.07, *p* = 0.15
	Mint	75.31	18.23	68.27	18.49	
	MB	66.31	18.16	67.63	15.21	
	ML	67.72	21.67	66.13	19.21	
	MO	70.18	18.32	64.09	14.20	
	Tests of between conditions	*F* (4, 210) = 9.102, *p* < 0.001*** Ctl vs. Mint: *p* < 0.001, 95% CI: [11.52, 29.65] Ctl vs. MB: *p* < 0.001, 95% CI: [6.71, 24.83] Ctl vs. ML: *p* < 0.001, 95% CI: [6.67, 24.79] Ctl vs. MO: *p* < 0.001, 95% CI: [6.87, 24.99]				
Refreshing sensation	Ctl	11.90	14.88	14.81	13.97	*F* (1, 210) = 13.51, *p* < 0.001*** Post Hoc: MW vs. R2: *p* = 0.005**, 95% CI: [−17.44, −3.14])
	Mint	63.77	22.87	43.40	25.40	
	MB	64.18	19.49	42.45	21.59	
	ML	60.40	22.15	42.40	22.80	
	MO	65.95	23.19	47.40	21.60	
	Tests of between conditions	*F* (4, 210) = 37.69, *p* < 0.001*** Ctl vs. Mint: *p* < 0.001, 95% CI: [30.99, 53.45] Ctl vs. MB: *p* < 0.001, 95% CI: [29.51, 51.98] Ctl vs. ML: *p* < 0.001, 95% CI: [30.63, 53.09] Ctl vs. MO: *p* < 0.001, 95% CI: [34.85, 57.32]				
Stimulative sensation	Ctl	7.36	13.35	9.22	13.46	*F* (1, 210) = 29.07, *p* < 0.001*** Post Hoc: MW vs. R2: *p* < 0.001***, 95% CI: [−22.73, −9.17]
	Mint	44.45	26.16	28.36	20.75	
	MB	52.40	21.66	28.63	25.12	
	ML	48.86	23.71	31.0	25.33	
	MO	54.27	21.41	30.36	24.03	
	Tests of between conditions	*F* (4, 210) = 18.55, *p* < 0.001*** Ctl vs. Mint: *p* < 0.001, 95% CI: [15.72, 40.50] Ctl vs. MB: *p* < 0.001, 95% CI: [19.83, 44.61] Ctl vs. ML: *p* < 0.001, 95% CI: [19.25, 44.02] Ctl vs. MO: *p* < 0.001, 95% CI: [21.63, 46.41])				
Cooling sensation	Ctl	26.59	20.65	26.72	22.38	*F* (1, 210) = 28.36, *p* < 0.001*** Post Hoc: MW vs. R2: *p* = 0.005, 95% CI: [−22.25, −8.03]
	Mint	76.04	16.36	61.72	20.58	
	MB	77.0	18.77	57.81	25.18	
	ML	73.22	20.49	63.81	20.53	
	MO	77.09	18.31	68.40	23.00	
	Tests of between conditions	*F* (4, 210) = 32.62, *p* < 0.001*** Ctl vs. Mint: *p* < 0.001, 95% CI: [28.33, 52.11] Ctl vs. MB: *p* < 0.001, 95% CI: [28.06, 51.84] Ctl vs. ML: *p* < 0.001, 95% CI: [26.15, 19.93] Ctl vs. MO: *p* < 0.001, 95% CI: [31.42, 55.21])				

Mint, peppermint; MB, peppermint + bergamot; ML, peppermint + lavender; MO, peppermint + sweet orange; Ctl, control condition.

**p < 0.01.

*** *p* < 0.001.

## 4 Discussions

This study investigated the psychophysiological effects of aromatic mouthwashes during the resilience phase after a short-term cognitive stressor, utilizing bio-signals as indicators. In this study, we hypothesized that the administration of aromatic mouthwashes will alleviate physiological stress responses within the ANS, with a greater decrease in HR and SCL and a greater increase in the HF component of HRV. The findings supported the expected working hypothesis by demonstrating a significantly greater reduction in HR in MB, ML, and MO mouthwashes compared to the Ctl condition, suggesting a potential suppression of the cardiac SNS during R2. Mint, MB, and MO mouthwashes demonstrated a significant increase in the HF component of HRV, indicating a suppression of cardiac PSNS activity. These findings imply that citrus mouthwashes such as MB and MO have the potential to alleviate the physiological stress response (in terms of cardiac activity) compared to the control condition. The results of the psychological assessment align with these physiological measures, indicating a positive subjective experience among participants using aromatic mouthwashes compared to the control condition. The integration of both bio-signals and psychological assessments in the study is pivotal for achieving a comprehensive understanding of an individual's response to aromatic mouthwashes, encompassing both physiological and psychological dimensions, enhancing the reliability and validity of study outcomes.

Prior research has consistently demonstrated the stress-alleviating properties of citrus essential oils (29, 32, 43, 44). In line with this premise, the present study findings revealed that mouthwash infused with orange essential oil and bergamot essential oil demonstrated effectiveness in resilience from acute stress response, as manifested by a reduction in HR and enhancement in the HF component of HRV during R2. These findings are consistent with previous study findings, where inhalation of mild orange essential oil influenced cardiac nervous system activity but had no significant effect on the peripheral nervous system (32), and the inhalation of bergamot essential oil showed a similar effect on PSNS activity, indicating stress-reducing effects (29). This implies the plausible psychophysiological impacts of orange and bergamot essential oils when administered through mouthwashes. Furthermore, Ishikawa et al. (15, 38) reported that the citrus mint mouthwash exhibited a greater ability to promote recovery from physiological stress response compared to the spice mint mouthwash (15), and the use of non- or low-alcohol mouthwashes promotes recovery from peripheral stress response (38). Such mouthwashes seem particularly suited for situations requiring relaxation, evident in the decrease in SCL during R2 (38). Similarly, another study, utilizing a wristwatch-type HR monitoring device, explored the effects of mouthwash use on sleep. It revealed that mint-flavoured mouthwash induced a relaxing effect, reflected in subjective scores and a positive mood before sleep (37). These findings collectively emphasize the potential therapeutic implications of specific mouthwash formulations, particularly those incorporating citrus essential oils, in managing stress and promoting relaxation.

The study's insights into the psychophysiological effects of specific aromas, such as peppermint, bergamot, sweet orange, and lavender, provide a foundation for developing tailored oral care formulations. This study which incorporated a variety of aromas in the mouthwash formulations introduced diverse flavours, stimulative sensations, and refreshing experiences to the users. This variation not only facilitated the recovery from acute stress responses but also provided participants with a range of sensory encounters throughout the experiment. Companies can leverage this knowledge to create products with carefully balanced aromatic profiles that maximize stress-alleviating properties. By integrating biological signals with psychological assessments, this study has expanded our understanding of mouthwashes, particularly their psychophysiological effects during post-acute stress resilience. This approach, encompassing both bio-signals and psychological parameters, enhanced the accuracy and depth of the research findings. The insights gained from this study offer potential avenues for the development of innovative oral care products that not only enhance oral health but also promote stress reduction. The incorporation of stress-alleviating aromas in oral care products opens the door for promoting stress reduction as part of daily hygiene routines.

However, it is essential to acknowledge certain limitations in this study. The intentional recruitment of a homogeneous sample comprising female university students aligns with several considerations related to the research objectives and the characteristics of the target market of the developed oral care product. This homogeneity can enhance the internal validity of the study by minimizing the influence of gender-related physiological and psychological differences. This deliberate selection might introduce biases into the results due to potential variations in gustation based on gender (45, 46) and different phases of the menstrual cycle (47), which could influence the outcomes. Additionally, it is worth noting that bio-signals such as SCL might exhibit fluctuations influenced by hormonal imbalances during various menstrual cycle phases in women (48). Moreover, it is noteworthy that all the mouthwashes utilized in this study were formulated with a base of peppermint essential oil. The specific combination and concentrations of these aromas could also contribute to the observed variations. Further research incorporating diverse aroma compositions could provide a more profound understanding of the effects of aromatic mouthwashes.

### 4.1 Future implications

In the future, the integration of bio-signals using wearable technology (37, 49), facial expression capturing (50), and other advanced physiological and psychological measurements (1) could offer a more comprehensive understanding of the intricate relationship between aromatic mouthwashes and physiological stress reduction. This could potentially lead to the development of personalized oral care interventions that cater to individual preferences and physiological responses, ultimately enhancing the overall well-being and oral health of users.

## 5 Conclusion

This study investigated the psychophysiological effects of aromatic mouthwashes in the resilience period following a short-term cognitive stressor. The experimental results suggested that rinsing with citrus-flavored mouthwashes has a positive impact in alleviating the physiological stress response (in terms of cardiac activity). These findings may have practical implications for the development of innovative, novel oral care products that promote stress reduction and improve oral health.

## Data Availability

The original contributions presented in the study are publicly available. This data can be found here: https://osf.io/j9n8t/ doi:10.17605/OSF.IO/J9N8T.
